# Reducing the Core Losses of Fe-Si-B Amorphous Alloy Ribbons by High Cooling Rate Planar Flow Casting

**DOI:** 10.3390/ma15030894

**Published:** 2022-01-25

**Authors:** Deren Li, Wenjun Wang, Tiancheng Liu, Lijun Li, Zhichao Lu

**Affiliations:** 1 School of Electrical Engineering, Beijing Jiaotong University, Beijing 100044, China; luzhch@bjtu.edu.cn; 2 CITIC Metal Co., Ltd., Beijing 100004, China; wangwj3@citic.com; 3 Advanced Technology and Materials Co., Ltd., China Iron and Steel Research Institute Group, Beijing 100086, China; liutiancheng@atmcn.com (T.L.); lilijun@atmcn.com (L.L.)

**Keywords:** planar flow casting, amorphous alloy ribbon, cooling rate, coercive force, core losses

## Abstract

In the planar flow casting (PFC) process, the cooling rate significantly affects the structure and properties of a cast ribbon. The influence of the thermal conductivity of the cooling wheel substrate on cooling rate was simulated by a numerical method, and it is shown that a higher thermal conductivity of the cooling wheel substrate leads to a higher cooling rate in the PFC process. Two copper-beryllium (Cu-2Be) rings with thermal conductivities of 175.3 W/m·K and 206.5 W/m·K were manufactured and installed onto a wheel core as the substrate of the cooling wheel. The effects of cooling rate on the soft magnetic properties of Fe-Si-B amorphous ribbons were investigated by pragmatic ribbon casting. The results show that the increment in the thermal conductivity of the cooling wheel substrate from 175.3 W/m·K to 206.5 W/m·K lowered the coercive force of amorphous ribbon from 2.48 A/m to 1.92 A/m and reduced the core losses at 1.4 T and 50 Hz by up to 22.1%.

## 1. Introduction

Fe-based amorphous ribbons play an important role in high-efficiency energy conversion devices, and have been widely used in the fields of electric power, power electronics and renewable energy as distribution transformers [1,2,3], medium-frequency inductors [4], reactors [5], and motors [6,7], etc. Fe-based amorphous ribbon materials are characterized as energy-saving materials, due to the one-step energy-saving production process of planar flow casting (PFC) [8], and the novel properties arising from their unique microstructure as well as the thin ribbon gauge [9]. Therefore, minor improvements in core losses could lead to considerable energy savings [10].

The PFC is a complicated non-equilibrium solidification process, where heat and mass transfer, multiphase fluid flow and solidification occur concurrently. The PFC process parameters can be divided into configuration parameters and casting process parameters. The configuration parameters mainly include nozzle configuration parameters, such as the slot width of the nozzle, inclination angle of nozzle, parallelism of the nozzle to the axis of the cooling wheel, etc., and cooling wheel configuration parameters such as the cooling wheel dimensions, thickness of the cooling wheel substrate and thermal conductivity of cooling wheel substrate, etc. The casting process parameters mainly include the temperature of molten alloy, applied pressure of the molten alloy to the bottom of the nozzle, the nozzle–wheel distance, velocity of cooling wheel substrate, and temperature and flow rate of coolant in the cooling wheel. PFC processes have been the subject of numerous studies by a number of scientists and engineers, with the emphases mainly focused on puddle formation [11,12] and process stability [13,14], as well as the influences of processing parameters and processing conditions on materials structure [15,16], ribbon thickness [17,18], and ribbon surface topography [19,20].

Fe-based amorphous ribbons exhibit low core losses due to their ultra-thin thickness of 20–30 microns, higher electrical resistivity of 100–130 μΩ·cm and lack of magneto-crystalline anisotropy. The demands of a high efficiency and energy saving pose a challenge to further reduce the core losses of amorphous ribbons. To date, numerous studies have been conducted on materials development [2,9,21], subsequent annealing and the structural design of magnetic cores [3,22] to further reduce the core losses of Fe-based amorphous materials, cores, and components. However, few reports are found on the influence of process parameters on core losses of Fe-based amorphous ribbon produced by the PFC process.

This work studies the influence of the thermal conductivity of cooling wheel substrates on the cooling rate of Fe-based amorphous ribbon and investigates the effects of cooling rate on the soft magnetic properties of Fe-Si-B amorphous ribbons.

## 2. Materials and Methods

### 2.1. Calculation Methods and Parameters

**Figure 1:**
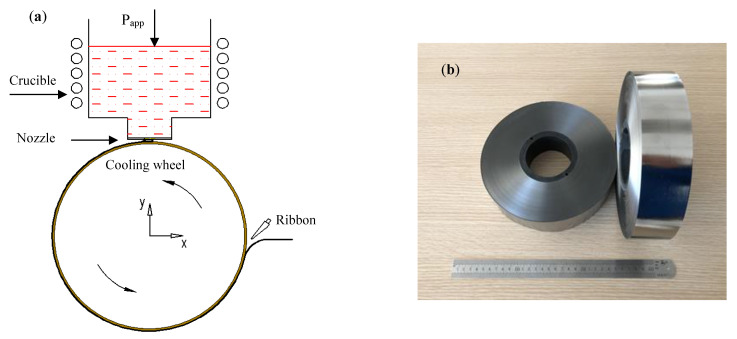
(**a**) The schematic geometry configurations with the corresponding coordinate system, where P_app_ is an applied pressure to the surface of molten metallic alloy. The coordinate origin is at the center of the cooling wheel and x = 0.0 m is aligned with the center of nozzle slot [23]. (**b**) The as-cast Fe-Si-B amorphous ribbons.

To analyze the influence of the thermal conductivity of the cooling wheel substrate on the cooling rate of molten alloy during the PFC process, the heat and mass transfer during the PFC process were simulated by ANSYS FLUENT software. Figure 1a shows the schematic geometry configurations with the corresponding coordinate system, where P_app_ is an applied pressure to the surface of molten metallic alloy, the coordinate origin is at the center of the cooling wheel and x = 0.0 m is aligned with the center of the nozzle slot. A detailed description of the physical model, solution method and boundary conditions is presented elsewhere [23]. The main calculation parameters employed for this simulation are listed as follows: the applied pressure at the nozzle bottom is 45 kPa, the cooling wheel outer diameter is 300 mm, the thickness of the Cu-2Be ring is 10 mm, the linear velocity of cooling wheel substrate surface is 20 m/s, the nozzle slot width is 0.3 mm, the nozzle wheel gap distance is 0.15 mm, the temperature of the molten alloy is 1420 °C, the initial temperature of the Cu-2Be substrate and circumferential air is 32 °C, and the glass formation temperature of the Fe-Si-B alloy is assumed to be 750 °C [12]. Figure 1b shows the Fe-Si-B amorphous ribbons prepared by PFC, employing the above geometry configuration and calculation parameters.

### 2.2. Experimental Methods

Two hot-rolled copper-beryllium (Cu-2Be) cylindrical rings were manufactured from the same wrought cylindrical ingot. The composition of the Cu-2Be rings is Be_1.87_Co_0.26_Ni_0.01_Fe_0.03_Al_0.02_Si_0.03_Cu_97.77_ (wt.%). The hot-rolled rings were firstly solution annealed at 785 °C with a soaking time of 3 h and then water quenched within 30 s in a water tank with water agitation. The aging treatments of the solution-annealed Cu-2Be rings were carried out at 335 °C and 380 °C with soaking times of 4 h and 6 h in a muffle furnace, respectively. The inner diameter, outer diameter and height of the machined cylindrical rings are 275 mm, 305 mm and 300 mm, respectively. The thermal conductivities of the two Cu-2Be rings are 175.3 W/m·K and 206.5 W/m·K, respectively. A cooling wheel system is composed of a stainless steel wheel core and a cylindrical ring, where the wheel core has axially extended water channels formed about its outer peripheral surface, and the cylindrical ring was shrink-fitted onto the wheel core. The outer diameter of the Cu-2Be ring on the cooling wheel was 300 mm after precision machining.

Alloy ribbons with a nominal composition of Fe_80_Si_9_B_11_ (at. %) were produced by using the Cu-2Be as the substrate of the cooling wheel. The ribbon width was 50 ± 0.5 mm and the ribbon thickness was 25 ± 0.5 μm.

XRD studies were carried out using a Bruker D8 Discover X-Ray diffractometer (Bruker in Berlin, Germany) with CuKα radiation. The 2θ Bragg angle was varied from 20° to 100°. The XRD measurements were carried out on the free surface of the as-cast ribbons.

The quasi-static hysteresis loops were measured using a MATS2010SD static magnetic property analyzer (LINK JOIN, Loudi, Hunan, China). The core losses and apparent power were measured using an IWATSU SY-8232 B-H analyzer (IWATSU, Tokyo, Japan). All the measured samples are toroidal cores, with an inner diameter of 30 mm, outer diameter of 35 mm, and height of 50 mm, subjected to heat treatment (Advanced Technology and Materials Co., Ltd., Beijing, China) at 365 °C for 60 min with an applied longitudinal field of 400 A/m.

## 3. Results and Discussion

### 3.1. Estimation of Cooling Rate of Solidified Alloy during PFC Process

**Figure 2:**
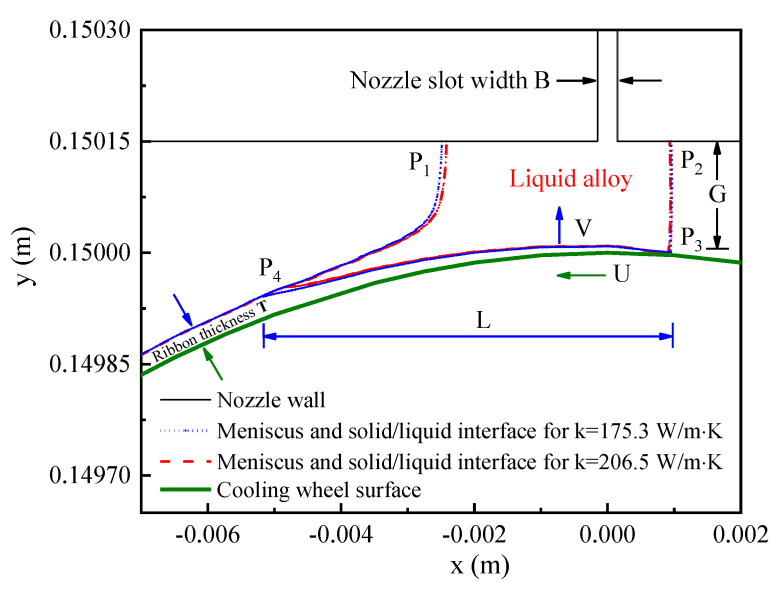
The simulation results of upstream meniscus, downstream meniscus, and growth of liquid/solid interface in quasi-static process for substrate thermal conductivities of 175.3 W/m·K and 206.5 W/m·K, where B is the slot width of the nozzle, G is the nozzle wheel distance, T is ribbon thickness, L is the puddle length, U is the linear velocity of moving substrate surface of cooling wheel, and V is the velocity of solidification front.

**Figure 3:**
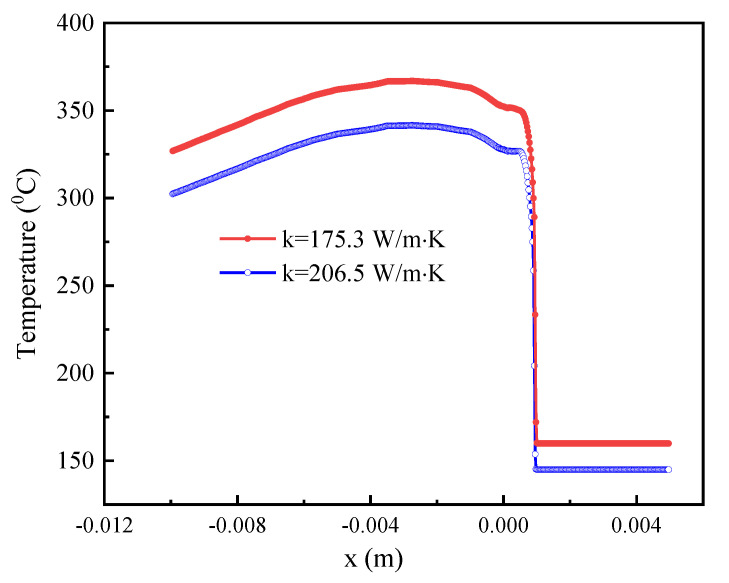
The calculation results of the interface temperature between the solidified alloy and Cu-2Be substrate surface in the puddle region for the Cu-2Be substrate with thermal conductivities of 175.3 W/m·K and 206.5 W/m·K at the 130th revolution. (Both viewed along Z-axis direction).

To estimate the influence of the thermal conductivity of the cooling wheel substrate on the cooling rate of solidified alloy in the puddle region, the PFC process was simulated and thermal conductivities of 175.3 W/m·K and 206.5 W/m·K were used for the Cu-2Be rings as a substrate of the cooling wheel. It has been shown that the puddle reaches a quasi-steady state after the 60th revolution for a cooling wheel with a diameter of 300 mm and Cu-2Be thickness of 10 mm [23]. In this work, the PFC process with 130 revolutions was used to estimate the quasi-steady state. Figure 2 shows the simulation results of the upstream meniscus, downstream meniscus, and growth of liquid/solid interface at the 130th revolution for Cu-2Be substrate with thermal conductivities of 175.3 W/m·K and 206.5 W/m·K, respectively. In Figure 2, upstream meniscus and downstream meniscus are plotted by the line of the volume fraction of melt = 1 (interface between air and liquid alloy), and the liquid/solid interface is plotted by the isothermal line of glass formation temperature T_g_ = 750 °C. P_1_ and P_2_ are the Tri-junction of air, liquid alloy and solid nozzle wall, P_3_ and P_4_ are the Tri-junction of air, liquid alloy and solid ribbon, T is the ribbon thickness, L is the puddle length from P_3_ to P_4_, U is the linear velocity of the moving substrate surface of the cooling wheel, and V is the velocity of the solidification front. Figure 3 shows the calculation results of the interface temperature, between the solidified alloy and Cu-2Be substrate surface in the puddle region, for the Cu-2Be substrate with a different thermal conductivity at the 130th cycle.

The growth of the liquid/solid interface is from P_3_ to P_4_ for this case. As shown in Figure 2, the solidification time is τ = L/U, the cooling rate is estimated by η = (T_g_ − T_s_)/τ, where T_s_ is the maximum interface temperature between the solidified alloy and Cu-2Be substrate surface in the puddle region. In order to calculate the corresponding cooling rate, data were extracted from the liquid/solid interface of the puddle and the interface temperature, between the solidified alloy and Cu-2Be substrate surface. Based on the extracted data listed in Table 1, it can be calculated that the cooling rates are 1.2 × 10^6^ K/s and 1.44 × 10^6^ K/s for thermal conductivities of k = 175.3 W/m·K and k = 206.5 W/m·K, respectively. The cooling rate increased by 14.3% due to the increment in substrate thermal conductivity. Therefore, the increase in thermal conductivity of cooling wheel substrate from k = 175.3 W/m·K to k = 206.5W/m·K resulted in the corresponding increase in the cooling rates from 1.2 × 10^6^ K/s to 1.44 × 10^6^ K/s, as shown by the calculation results.

### 3.2. Effect of Cooling Rate on Soft Magnetic Properties of Amorphous Ribbons

**Figure 4:**
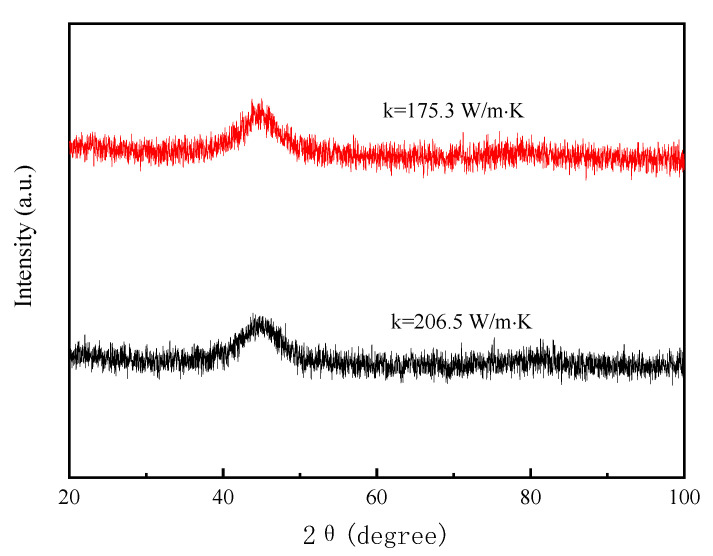
XRD patterns of the as-cast Fe-Si-B ribbons produced by cooling wheels with substrate thermal conductivities of 175.3 W/m·K and 206.5 W/m·K.

To alter the cooling rate of solidified alloy in the puddle region during the PFC process, two Cu-2Be rings with thermal conductivities of 175.3 W/m·K and 206.5 W/m·K were installed in sequence onto the wheel core, and Fe-Si-B amorphous ribbons were produced by PFC. Figure 4 shows the XRD patterns of the as-cast Fe-Si-B ribbons produced by the cooling wheels, with substrate thermal conductivities of 175.3 W/m·K and 206.5 W/m·K, respectively. It can be seen from the figure that both XRD patterns consist of only broad peaks without significant crystalline peaks, indicating that both the Fe-Si-B alloy ribbons are composed of an amorphous phase.

**Figure 5:**
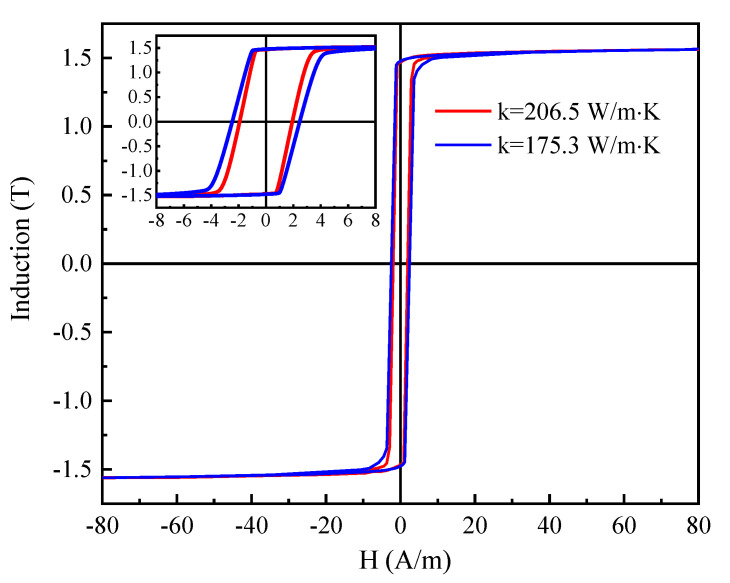
The quasi-static hysteresis loops of Fe-Si-B ribbons produced by cooling wheel with different substrate thermal conductivities and subjected to annealing at 365 °C for 60 min with an applied longitudinal field of 400 A/m.

Figure 5 shows the quasi-static hysteresis loops of Fe-Si-B ribbons produced by the cooling wheel with different substrate thermal conductivities and then annealed at 365 °C for 60 min with an applied longitudinal field of 400 A/m. Although the magnetic flux density at 80 A/m has the same value of B_80_ = 1.56 T for both ribbons, the coercive forces of the annealed ribbons are different, where the higher thermal conductivity value of 206.5 W/m·K of the cooling wheel substrate corresponds to a lower coercive force value, Hc = 1.92 A/m, and the lower thermal conductivity value 175.3 W/m·K of the cooling wheel substrate corresponds to a higher coercive force value H_c_ = 2.48 A/m. Therefore, the increase in thermal conductivity of the cooling wheel leads to a decrease in the hysteresis loss of the annealed ribbon due to the reduction in coercive force.

**Figure 6:**
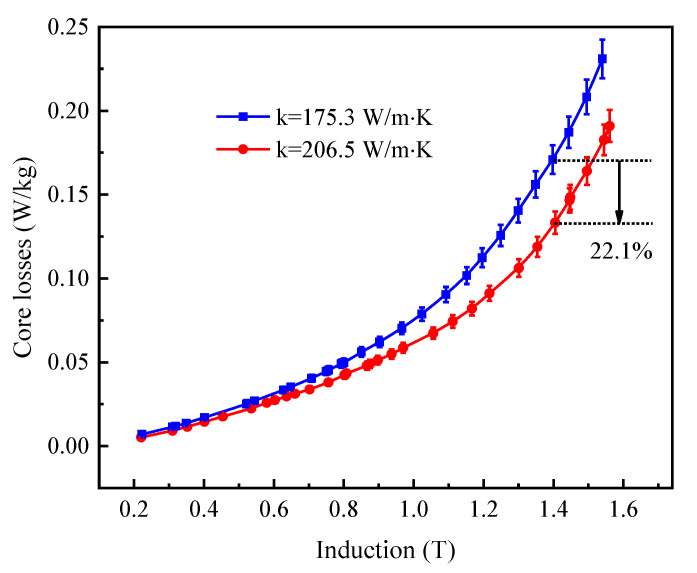
The core losses at 50 Hz as a function of flux density for Fe-Si-B ribbons produced by cooling wheel with different substrate thermal conductivities and subjected to annealing at 365 °C for 60 min with an applied longitudinal field of 400 A/m.

**Figure 7:**
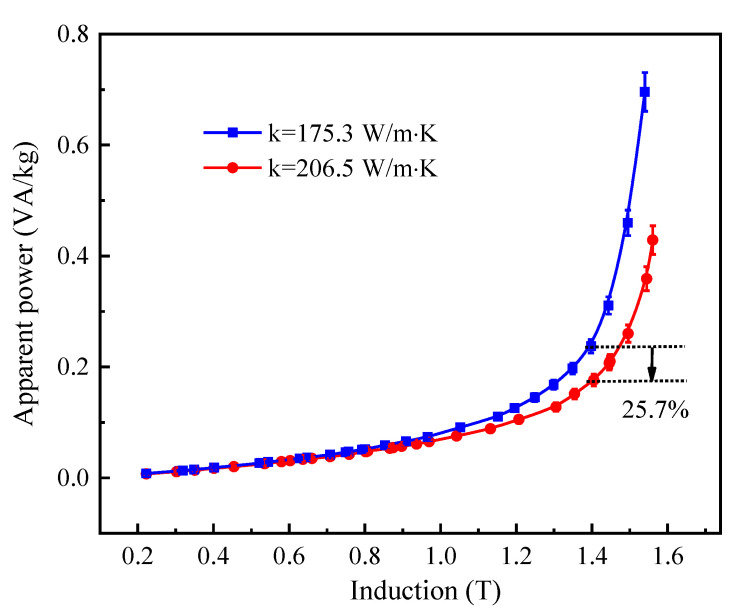
The apparent power at 50 Hz as a function of flux density for Fe-Si-B ribbons produced by cooling wheel with different substrate thermal conductivities and subjected to annealing at 365 °C for 60 min with an applied longitudinal field of 400 A/m.

Figure 6 and Figure 7 show the core losses and apparent power at 50 Hz as a function of flux density for Fe-Si-B ribbons produced by the cooling wheel with different substrate thermal conductivities and subjected to annealing at 365 °C for 60 min with an applied longitudinal field of 400 A/m. The typical values of core losses and apparent power of annealed Fe-Si-B ribbons at 50 Hz and 1.4 T are summarized in Table 2. The increase in thermal conductivity of the cooling wheel substrate from 175.3 W/m·K to 206.5 W/m·K resulted in a decrease in core losses from 0.1708 W/kg to 0.1331 W/kg. Meanwhile, the apparent power was reduced from 0.2374 VA/kg to 0.1762 VA/kg. As the thermal conductivity increased from 175.3 W/m·K to 206.5 W/m·K, the core losses and apparent power were reduced by up to 22.1% and 25.7% at 50 Hz and 1.4 T, respectively. Thus, it can be concluded that an increase in thermal conductivity of the cooling wheel substrate leads to an increase in the cooling rate of solidified alloy in the PFC process, which results in the improvement of soft magnetic properties such as lower coercive force, lower core losses and lower apparent power.

## 4. Conclusions

The influence of the thermal conductivity of the cooling wheel substrate on the cooling rate in the PFC process was simulated by a numerical method, and the results show that the increase in the thermal conductivity of the cooling wheel substrate from 175.3 W/m·K to 206.5 W/m·K led to an increase in cooling rate from 1.2 × 10^6^ K/s to 1.44 × 10^6^ K/s during the PFC process. The effect of cooling rate on the soft magnetic properties of Fe-Si-B amorphous ribbons was investigated by practical ribbon casting, using cooling wheels with different thermal conductivities. It was revealed that the increase in the thermal conductivity of cooling wheel substrate from 175.3 W/m·K to 206.5 W/m·K in the PFC process resulted in a decrease in the coercive force of amorphous ribbon from 2.48 A/m to 1.92 A/m, and the reduction in core losses and apparent power at 1.4 T and 50 Hz up to 22.1% and 25.7%, respectively. This work provides an effective method of reducing the core losses of Fe-Si-B amorphous ribbons by means of increasing the cooling rate in the PFC process.

## Figures and Tables

**Table 1:** The coordinates of liquid/solid interface and the interface temperature between the solidified alloy and Cu-2Be substrate surface in the puddle region.

K (W/m·K)	P_3x_ (mm)	P_4x_ (mm)	L = P_3x_ − P_4x_ (mm)	T_s_ (°C)
175.3	0.96	−5.18	6.08	366.5
206.5	0.92	−4.76	5.68	341.2

**Table 2:** The cooling wheel condition and typical values of core losses and apparent power of Fe-Si-B ribbons subjected to annealing at 365 °C for 60 min with an applied longitudinal field of 400 A/m.

Thermal Conductivity of Cooling Wheel (W/m·K)	Core Losses at 50 Hz and 1.4 T (W/kg)	Apparent Power at 50 Hz and 1.4 T (VA/kg)
206.5	0.1331	0.1762
175.3	0.1708	0.2374

## Data Availability

The datasets analyzed and/or generated during the current study are available from the corresponding author on reasonable request. The data presented in this study are available upon request from the corresponding author.
